# Site‐specific distribution of oak rhizosphere‐associated oomycetes revealed by cytochrome c oxidase subunit II metabarcoding

**DOI:** 10.1002/ece3.5577

**Published:** 2019-08-16

**Authors:** Melanie Sapp, Nicolas Tyborski, Anja Linstädter, Aida López Sánchez, Tim Mansfeldt, Guido Waldhoff, Georg Bareth, Michael Bonkowski, Laura E. Rose

**Affiliations:** ^1^ Cluster of Excellence on Plant Sciences (CEPLAS) Population Genetics Heinrich Heine University Düsseldorf Germany; ^2^ Botanical Institute, Range Ecology and Range Management University of Cologne Cologne Germany; ^3^ Institute of Crop Science and Resource Conservation (INRES) University of Bonn Bonn Germany; ^4^ Departamento de Sistemas y Recursos Naturales Universidad Politécnica de Madrid Madrid Spain; ^5^ Institute of Geography University of Cologne Cologne Germany; ^6^ Cluster of Excellence on Plant Sciences (CEPLAS) Institute of Zoology Terrestrial Ecology University of Cologne Cologne Germany

## Abstract

The phylum Oomycota comprises important tree pathogens like *Phytophthora quercina*, involved in central European oak decline, and *Phytophthora cinnamomi* shown to affect holm oaks among many other hosts. Despite the importance to study the distribution, dispersal and niche partitioning of this phylum, metabarcoding surveys, and studies considering environmental factors that could explain oomycete community patterns are still rare. We investigated oomycetes in the rhizosphere of evergreen oaks in a Spanish oak woodland using metabarcoding based on Illumina sequencing of the taxonomic marker cytochrome c oxidase subunit II (cox2). We developed an approach amplifying a 333 bp long fragment using the forward primer Hud‐F (*Mycologia*, 2000) and a reverse primer found using DegePrime (*Applied and Environmental Microbiology*, 2014). Factors reflecting topo‐edaphic conditions and tree health were linked to oomycete community patterns. The majority of detected OTUs belonged to the Peronosporales. Most taxa were relatives of the Pythiaceae, but relatives of the Peronosporaceae and members of the Saprolegniales were also found. The most abundant OTUs were related to *Globisporangium irregulare* and *P. cinnamomi*, both displaying strong site‐specific patterns. Oomycete communities were strongly correlated with the environmental factors: altitude, crown foliation, slope and soil skeleton and soil nitrogen. Our findings illustrate the significance of small scale variation in habitat conditions for the distribution of oomycetes and highlight the importance to study oomycete communities in relation to such ecological patterns.

## Graphical abstract

The phylum Oomycota comprises important tree pathogens, but knowledge on environmental factors that could explain their community patterns is scarce. To advance our understanding, oomycetes in the oak rhizosphere were studied using metabarcoding of the taxonomic marker cytochrome c oxidase subunit II and linked to biotic and abiotic variables. Oomycete communities were strongly correlated with the environmental factors altitude, crown foliation, slope and soil skeleton and soil nitrogen.

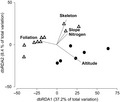

## 1 INTRODUCTION

Members of the phylum Oomycota are globally distributed and adapted to a large variety of ecosystems (Thines, 2014). Although oomycetes display a great variety of lifestyles, it is thought that they are largely adapted to a pathogenic lifestyle given the high number of known pathogens (Beakes, Glockling, & Sekimoto, 2012; Thines, 2014; Thines & Kamoun, 2010). The phylum contains many well‐known plant pathogens including the causative agent of sudden oak death, *Phytophthora ramorum* (Grünwald, Garbelotto, Goss, Heungens, & Prospero, 2018), and *P. quercina* infecting European oaks (Jönsson, 2004; Jung, Cooke, Blaschke, Duncan, & Oßwald, 1999). However, despite their importance for agriculture and forestry, the relationship between niche partitioning, oomycete community structure, host distribution, and environment is poorly understood.

Oomycetes have been shown to play key roles in European oak decline, affecting native oak species across the continent. This syndrome can be divided into two geographic groups: (a) central European and (b) Mediterranean or Iberian oak decline (Brasier, 1996). The latter affects silvo‐pastoral systems dominated by the keystone species cork and holm oak (*Quercus suber* and *Q. ilex* respectively) (Bugalho, Caldeira, Pereira, Aronson, & Pausas, 2011). These unique ecosystems are sustained by human activity called “Dehesa” in Spain or “Montado” in Portugal. Iberian oak decline is characterized by a reduction of fine root density accompanied by root lesions leading to wilting and discoloration of leaves and ultimately to a reduction in canopy density (Corcobado, Cubera, Moreno, & Solla, 2013; de Sampaio e Paiva Camilo‐Alves, Clara, & Almeida Ribeiro, 2013). In addition to abiotic factors such as water stress, oak pathogens such as *Phytophthora cinnamomi* are thought to predispose the trees to decline (Brasier, 1996; Brasier, Robredo, & Ferraz, 1993; Corcobado, Cubera, et al., 2013; Moricca et al., 2016; de Sampaio e Paiva Camilo‐Alves et al., 2013). However, interactions between evergreen oaks and pathogens appear more complex. These trees are colonized by a broad range of primary and opportunistic pathogens (Moricca et al., 2016) of which the oomycete genera *Phytophthora* and *Pythium* are consistently found in combination with root damage (Jung, Blaschke, & Oßwald, 2000; Lehtijärvi, Aday Kaya, Woodward, Jung, & Doğmuş Lehtijärvi, 2017; Romero et al., 2007). A rich diversity of *Phytophthora* species was recently described in soil sampled close to Spanish *Q. ilex* trees (Català, Berbegal, Pérez‐Sierra, & Abad‐Campos, 2017; Pérez‐Sierra et al., 2013). Surprisingly, among the *Phytophthora* species detected were *P. quercina*, known for its involvement in the central European decline, and a novel species (taxon ballota) (Català et al., 2017). However, when the effect of *Phytophthora* species on seedling growth of *Q. ilex* was tested, *P. quercina* did not display as severe effects as compared to *P. cinnamomi*, but it did show strong pathogenicity indicated by a McKinney index over 60 (Pérez‐Sierra et al., 2013). *Phytophthora cinnamomi* was also detected in *Q. suber* stands in Italy irrespective of decline symptoms (Scanu et al., 2013). These findings illustrate the complex interaction between potential oomycete pathogens and their oak hosts.

The majority of studies designed to detect Oomycetes involved in the decline have used cultivation based techniques and detected a high site variability for targeted pathogens (Duque‐Lazo, van Gils, Groen, & Navarro‐Cerrillo, 2016; Jung et al., 2018; Linaldeddu, Scanu, Maddau, & Franceschini, 2014; Pérez‐Sierra et al., 2013; Romero et al., 2007). For example in south‐western Spain and southern Portugal, sometimes the pathogens, *P. cinnamomi* and *Globisporangium spiculum,* coexisted, while at other sites, only one pathogen dominated (Romero et al., 2007). Similarly in Italy, *Phytophthora* species, *P. cinnamomi*, *P. cryptogea,* and *P. gonapodyides,* were detected in roots or soil surrounding holm oaks in only 50% of the studied sites, although the incidence of decline was high in nearly all plots studied (Linaldeddu et al., 2014). This raises the question what environmental factors are responsible for the observed differences in oomycete distribution.

The symptoms of Iberian oak decline, that is, a massive reduction in canopy density, are strongly linked to environmental factors (de Sampaio e Paiva Camilo‐Alves et al., 2013). In their synthesis, the authors report that soil characteristics linked to water stress, including compaction and soil depth, were the most important factors related to decline. This was followed by soil texture, soil nutrients, and topography. Exposition, soil type, and the presence of *P. cinnamomi* had smaller effects. However, the study could not resolve whether other oomycete taxa might contribute to decline symptoms.

Less is known on environmental factors explaining oomycete patterns and their potential use in predicting pathogen spread. In an experiment using cultivation techniques, the spatial distribution of *P. cinnamomi* and *G. spiculum* was linked to abiotic and biotic environmental variables including soil texture and shrub diversity (Gómez‐Aparicio et al., 2012). The relevance of these factors differed depending on forest type, but also on pathogen species. Pathogen abundance was negatively correlated with sand content, fitting well with the notion that *P. cinnamomi* requires humid conditions to spread (Hardham & Blackman, 2018). A more holistic view on environmental control of oomycete communities could be gained using cultivation‐independent methods.

Metabarcoding of micro‐eukaryotic communities has received increased attention in the last few years. For oomycete communities, the ITS1 fragment is commonly used (Agler et al., 2016; Riit et al., 2016; Sapp, Ploch, Fiore‐Donno, Bonkowski, & Rose, 2018; Vettraino, Bonants, Tomassini, Bruni, & Vannini, 2012) but also fragments of the 18S rDNA have been applied (Singer et al., 2016). A positive aspect of using markers like ITS1 for this purpose is the broad coverage of diversity in reference databases (Robideau et al., 2011). However, some species of relevant genera like *Bremia* are not sufficiently distinguished by this marker (Choi et al., 2017). Also, sequence alignments can be challenging due to large insertions for some oomycete taxa (Choi et al., 2015). Mitochondrial genes have been successfully used to provide good taxonomic resolution and robust evolutionary relationships between oomycete taxa. Furthermore, in combination with ITS, the use of the cytochrome c oxidase subunit II (cox2), in contrast to subunit I (cox1), resulted in good taxonomic resolution (Choi et al., 2015; Martin & Tooley, 2003). It is therefore not surprising that many researchers have adopted cox2 to infer phylogenetic relationships within the Peronosporales (Hudspeth, Nadler, & Hudspeth, 2000; Martin, 2000; Martin, Blair, & Coffey, 2014; Ploch et al., 2011; Thines et al., 2008; Uzuhashi, Tojo, & Kakishima, 2010). Although the cox2 product size varies between different taxonomic groups (Choi et al., 2015), it does not display the length polymorphism of ITS. However, the fragments currently employed for the description of oomycete isolates are generally too long for metabarcoding on commonly used platforms; thus, we developed an assay combining the primer Hud‐F (Hudspeth et al., 2000) and a new reverse primer found using DEGEPRIME (Hugerth et al., 2014) to shorten the read length, but retain sufficient resolution for taxonomic assignments. We used this cox2 metabarcoding approach to study the distribution of oomycetes in the rhizosphere of holm and cork oaks in relation to Iberian decline symptoms and linked oomycete community structure with habitat characteristics.

While some environmental factors favouring the spread of particular oomycete pathogens are well described, the factors determining the taxonomic distribution of oomycetes across habitats and communities have not been elucidated. In contrast, the factors structuring communities for other terrestrial micro‐eukaryotic groups are better understood. For example, fungal communities are structured mainly by environmental factors like pH (Glassman, Wang, & Bruns, 2017) and the community similarity in host‐associated fungi decays more slowly with distance than for free‐living fungi (Goldmann et al., 2016). The latter are known to vary in community structure on small spatial scales (Green et al., 2004), a circumstance so far unstudied at the oomycete phylum level. Our study aimed to fill this gap by analyzing oak rhizosphere oomycetes and relating their distribution to host and other biotic factors as well as abiotic habitat characteristics at low spatial scales. Such information is valuable not only to predict the spread of single taxa, but potentially for entire oomycete communities.

## 2 MATERIALS AND METHODS

### 2.1 Study area and sampling design

Field data were collected in a Mediterranean oak woodland on the farm “Dehesa San Francisco” in Andalusia, Spain. The climate is continental Mediterranean, with hot summers and precipitation concentrated in the spring and fall. Parent material of soil formation on the farm is schist. Due to a strongly dissected topography, colluvic material is found at footslopes. Predominantly, continuous rock (schist) starts ≤25 cm from the soil surface, and soils belong to the reference soil group (RSG) of leptosols (IUSS Working Group WRB 2014). Appropriate principal qualifiers for this RSG are lithic (continuous rock starts ≤10 cm from the soil surface), skeletic (having >40 vol% coarse fragments to continuous rock), Cambic (showing pedogenetic alteration), and Dystric (a base saturation <50%). On a small spatial scale, Cambisols (soils showing pedogenetic alteration) occur, and stagnic properties due to temporary water saturation are present in some areas. The latter is caused by soil compaction and interflow in the slopes. On these shallow, infertile soils, the tree cover is dominated by two evergreen oak species, that is, holm oaks and cork oaks. The low shrub layer is dominated by evergreen xerophytes (e.g., *Cistus salvifolius* L., *Cistus ladanifer* L., *Lavandula stoechas* Lam., and *Genista hirsuta* Vahl).

**Figure 1:**
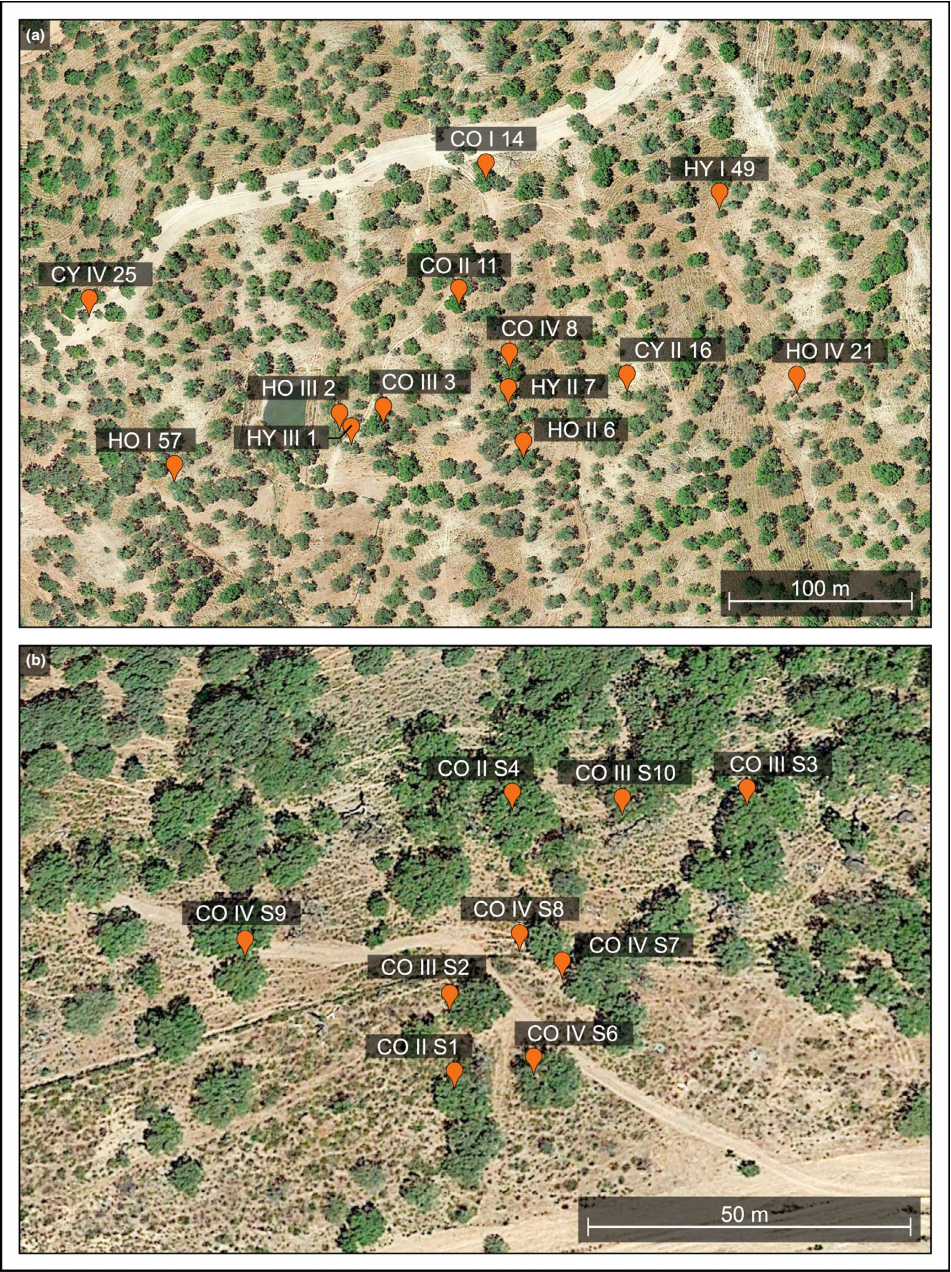
Overview of sampled trees. (a) Sampling in March 2016 (site 1), (b) Sampling in November 2016 (site 2). Tree labels indicate species affiliation (H = holm oak, C = cork oak) and a health status class (I = healthy trees with 100%–95% foliation; II = trees of an intermediate health and a foliation of 94%–80%; III = diseased trees with crown foliation of 79%–50%, and IV = severely diseased or dead trees with 49%–0% foliation) (Satellite imagery © 2017 Google Earth)

A total of 22 holm and cork oaks differing in size and vitality were selected for rhizosphere oomycete analyses. Sampling took place in March and November 2016 at two sites. These differed mainly in their exposition and tree species composition. While site 1 harbored both holm and cork oaks, site 2 was dominated by the latter. Specific locations of collected samples and sample IDs are shown in Figure 1 and Table S2.

### 2.2 Biotic characteristics

For each tree, crown foliation was used as proxy for tree health and was assessed as described previously (Müller & Stierlin, 1990). Here, crown foliation of 100% means fully foliated and 0% dead. In addition, health categories were established according to crown foliation: Trees with 100%–95% foliation were assigned to class I, and trees with foliation of 94%–80% were assigned to class II, whereas class III and class IV consisted of trees with crown foliation of 79%–50% or 49%–0%, respectively. To capture shading effects of tree canopies on subcanopy habitats (Linstädter, Bora, Tolera, & Angassa, 2015), we used the proxy “tree vigor.” This factor combines crown foliation and four variables characterizing a tree's spatial dimensions namely diameter at breast height (dbh), tree height, crown height, and radius (Voelker, Muzika, & Guyette, 2008). We measured dbh by tape to the nearest centimeter, while tree height, crown height, and crown diameter were measured to the nearest decimeter using a range pole. Crown radius was obtained by taking two perpendicular measurements of crown diameter that were subsequently averaged and converted to radius. Other biotic variables were measured within a 50 × 50 cm plot located at a distance of 1 m to the trunk in south‐western direction. These measurements were performed in the same growth period for which oomycete communities were sampled (growth period 2015/16 for site 1 and growth period 2016/17 for site 2). To characterize structural vegetation properties, we visually estimated the percentage cover of litter and bare soil following standard procedures (Linstädter et al., 2014). Plant litter decomposition was measured with the tea bag index (TBI) method (Keuskamp, Dingemans, Lehtinen, Sarneel, & Hefting, 2013). Field incubations were initiated in March of the respective growth period by placing pairs of green tea and rooibos tea bags at a depth of 8 cm in each plot. Tea bags were retrieved after 65–69 days; adhered soil particles were removed, and the mass remaining after oven drying was measured. Subsequently, the litter decomposition rate *k* (TBI_k) and the litter stabilization factor *S* (TBI_S) were calculated as described previously (Keuskamp et al., 2013). We assessed the floristic composition of each plot's herbaceous layer in mid‐May (end of growth period) by recording all vascular plant species within the respective plot and estimated each species’ ground cover. Herbarium specimens were taken for all plant species that could not be identified in the field for later identification. Taxonomic nomenclature follows The Plant List (http://www.theplantlist.org/). Based on floristic data, the Shannon diversity index V (Shannon, Petigara, & Seshasai, 1948) was calculated for subcanopy vegetation (Shannon(V)) to allow comparisons between plant and oomycete beta‐diversity as shown for other microbial groups (Prober et al., 2015).

### 2.3 Abiotic characteristics

To capture topo‐edaphic conditions, the specific geographic location of each tree was identified along with its altitude, slope, and aspect. Tree locations were georeferenced with accurate GPS measurements (<3 cm) using an established base station. Slope, aspect, and radiation received during a hydrological year (Rwt_HY) were computed from a digital elevation model (DEM) in ultra‐high spatial resolution (<3 cm). The DEM was derived from RGB imagery captured with an unmanned aerial vehicle (UAV). The UAV‐derived data were photogrammetrically processed in the software PhotoScan using structure from motion (SFM) and multi‐view stereopsis (MVS) methods (Harwin & Lucieer, 2012). Orthophotos and DEMs are a result of this software analysis (Bendig et al., 2015). Slope and aspect were then computed from the UAV‐derived DEM using the geographical information system (GIS) software ArcGIS.

These parameters were derived from an unmanned aerial vehicle (UAV)‐borne digital surface model (DSM), which was created from orthophotos using structure from motion (SfM) (Bendig et al., 2015). The slope and aspect calculations were done with ArcGIS.

Soil was sampled at a distance of 1 m from the trunk and a soil depth of around 5–20 cm alongside roots. The soils were manually homogenized in the field, sieved <2 mm, and air‐dried. The sieve residue equals the soil skeleton content (<2 mm). After transportation to Germany, the samples were stored at room temperature in the dark. Subsamples were ground to analytical grain size with a grinding jar and balls of yttrium‐partially stabilized zirconium oxide (MM400, Retsch). The soil parameters pH, nitrogen (N), carbon (C), C/N ratio, soil type, and skeleton were determined to link abiotic factors to oomycete communities. Soil pH was measured potentiometrically with a glass electrode in a 0.01 M CaCl_2_ solution mixed 5:1 with soil (v/v). Total carbon (C) and nitrogen (N) were determined on ground samples by dry combustion with a CNS analyzer (Vario EL cube, Elementar). Because the soils are free of inorganic carbon, TC equals organic carbon (OC). An overview of all samples with corresponding environmental variables can be found in Table S2.

### 2.4 Rhizosphere oomycete community profiling

Three roots per tree were sampled using sterile forceps around the same position (area of 1 × 1 m) as soil samples as described previously (Moreira & Martins, 2005). Samples were stored at −20°C for 4 days until reaching the laboratory, where they were stored at −80°C prior to DNA extraction.

Nucleic acids were extracted from the oak roots using the PowerPlant® RNA Isolation Kit with DNase (MO BIO Laboratories, Inc.). Root tissues were disrupted in a Tissue‐Lyser II (Qiagen) for 1 min at 30 Hz, following the addition of a 5 mm stainless steel bead per sample. After centrifugation, the root material was incubated at 25°C for 10 min in PR1 combined with Phenolic Separation Solution, with regular mixing. Samples were processed as suggested by the manufacturer, although the DNAse step was omitted. Nucleic acid quality was evaluated using the spectrophotometer DS‐11 FX (DeNovix) and on 0.8% agarose gels. Oomycete communities were analyzed based on a barcode region of the cytochrome c oxidase subunit II (cox2).

#### 2.4.1 Primer design for cox2 metabarcoding

**Figure 2:**
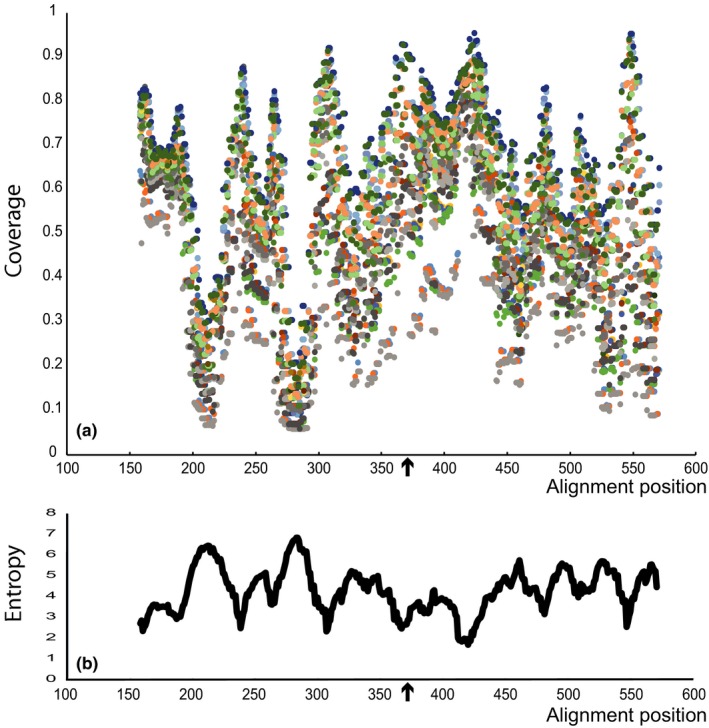
Sequence coverage and entropy for cox2 primers designed by DegePrime based on a trimmed alignment of 3,503 oomycete cox2 sequences. Shown is the sequence coverage of primers along the alignment length with varying degeneracy (1–20) and length (18, 20, 22). Each degeneracy and length combination is displayed in a different color (a). The corresponding entropy for each window position (b). Arrows indicate the position of the chosen reverse primer cox2_233D8r

Available cox2 sequences of oomycetes were downloaded from NCBI (Coordinators, 2015) on October 5, 2015. These were aligned using muscle 3.8.31 (Edgar, 2004), and sequences shorter than 500 bp were removed, leaving 3,503 sequences. The sequences were positioned at 138 bp and 620–656 bp using *Phytophthora infestans* (AY898628) as reference. Coverage of the sequence database encompassed major orders namely Albuginales, Anisolpidiales, Haptoglossales, Lagenidiales, Leptomitales, Myzocytiopsidales, Olpidiopsidales, Peronosporales, Rhipidiales, Saprolegniales, and Sclerosporales. In detail, 495 different species of 54 genera were included (Table S1). Using DegePrime (https://github.com/EnvGen/DegePrime (Hugerth et al., 2014)), we analyzed the aligned sequences for conserved and highly variable regions. The sequence alignment was further trimmed with a minimum cut‐off of 0.9 resulting in a database of sequences with a length of 471 bp. We tested different primer lengths (18, 20 and 22 bp) as well as degeneracies (D) from 1 to 20 to find suitable candidate primer sequences. These were determined based on high proportion of matched sequences and low entropy (Figure 2). Sequence coverage of primers along the alignment ranged from 95.5% to 5.8% at positions 424 (D16‐D20) and 143–147 (D1), respectively (Figure 2). Characteristics of candidate primers were analyzed using OligoCalc (Kibbe, 2007) following the guidelines outlined previously (Burpo, 2001). Also, self‐annealing with Illumina adapters was assessed. The best candidate cox2_233D8r (5′‐ GAATATTCATARSTCCARTACC −3′) at position 371 (*P. infestans*, AY898628) matched 79% of all sequences in the alignment. Matched and unmatched species are indicated in Table S1. The forward primer Hud‐F (Hudspeth et al., 2000) was used (Cox2hud‐F: 5′‐GGCAAATGGGTTTTCAAGATCC‐3′) together with the best candidate primer, resulting in a fragment of 333 bp.

#### 2.4.2 PCR conditions and validation

Both primers, Cox2hud‐F and cox2_233D8r, were amended with Illumina overhang adapters (Illumina's 16S Metagenomic Sequencing Library Preparation protocol). All PCR reactions were performed in triplicate using the Q5 High‐Fidelity PCR Kit (New England Biolabs) and contained 1x master mix, 0.3 μM of each primer, and 6–20 ng of template DNA. The final reaction volume of 20 μl was reached with nuclease‐free water (New England Biolabs). Amplification was carried out on a T100 thermal cycler (BioRad). Initial tests for best performing annealing temperatures were carried out using *Phytophthora infestans* strain T30‐4 (Haas et al., 2009). Following an initial denaturing step at 98°C for 2 min, 35 PCR cycles (consisting of denaturation at 98°C for 20 s, annealing at 58, 56, 54 or 52°C for 45 s, and extension at 72°C for 60 s) were run, followed by a final extension at 72°C for 5 min. Good results were obtained for most annealing temperatures. 54°C was chosen and successfully tested on other taxa, namely *Achlya racemosa* (CBS 108.35), *Salisapilia sapeloensis* (CBS 127,946), *Saprolegnia ferax* (CBS 305.37), *P. cinnamomi* (CBS 378.61), *Elongisporangium undulatum* (CBS 157.69), *Apodachlya brachynema* (CBS 184.82), *Halophytophthora exoprolifera* (CBS 252.93), and *Globisporangium sylvaticum* (CBS 720.94). To test the performance of the primer combination, cox2 amplicons of a mock community, consisting of the aforementioned taxa in equimolar concentrations, were sequenced alongside the samples as well as pooled negative PCR controls and a negative control index PCR reaction. For some rhizosphere samples, no PCR amplicons were obtained, including the samples from HYIII1, COIII3, HOI57, COIIIS3, COIIS4, and COIVS7 subsequently omitted from further analyses. Samples were prepared for sequencing as described previously (Sapp et al., 2018). Paired‐end sequencing was carried out by the BMFZ (Biologisch‐Medizinisches Forschungszentrum) of Heinrich Heine University Düsseldorf on an Illumina MiSeq machine using 5% PhiX. Index combinations used can be found in Table S3.

#### 2.4.3 Bioinformatic analyses

Sequencing resulted in 1,232,979 merged cox2 reads (Table S3). Sequence analyses were carried out as described previously (Sapp et al., 2018). Briefly, demultiplexed sequences were joined using fastq‐join (Aronesty, 2011) with minimum overlap of 50 bp. Primer trimming was carried out using QIIME version 1.9.1 (Caporaso et al., 2010) followed by quality filtering, clustering, and taxonomy assignment via USEARCH v9.2.64. Quality filtering consisted of applying a minimum length of 200 bp and a maximum error threshold of 1 (Edgar & Flyvbjerg, 2014). The reads were clustered at 97% sequence similarity using UPARSE (Edgar, 2013) followed by taxonomy assignment via sintax (Edgar, 2016) applying a cut‐off of 0.7 using the cox2 database described for primer development after the addition of decoy sequences covering plants and fungi.

After subtraction of non‐oomycete taxa, additional quality checks using sequence alignments against the reference sequences and removal of OTUs with low abundance (0.1% or lower) as suggested previously (Nelson, Morrison, Benjamino, Grim, & Graf, 2014), 21 OTUs were recovered. Following the rarefaction to 556 sequences, four metrics of alpha diversity were calculated: chao 1 (Chao, 1984), number of observed OTUs, Goods coverage (Good, 1953), and Shannon diversity index (Shannon et al., 1948).

Phylogenetic analyses were carried out using MEGA 7 (Kumar, Stecher, & Tamura, 2016). Representative sequences of OTUs were supplemented with sequences of close relatives identified during sintax taxonomy assignment and BLAST search (Johnson et al., 2008). A neighbor‐joining tree was constructed using the Tamura 3‐parameter method assuming a gamma distribution providing final taxonomic assignments for the obtained OTUs.

### 2.5 Statistical analyses

Differences in community patterns were assessed based on log‐transformed relative abundances of OTUs in relation to proxies for tree vitality, geographical location, soil properties, and other abiotic and biotic environmental variables. The following analyses were conducted: principal component analysis to gain a general overview of community patterns, PERMDISP (Anderson, Ellingsen, & McArdle, 2006) to test homogeneity of dispersions, ANOSIM and PERMANOVA (Anderson, Gorley, & Clarke, 2008) to identify environmental factors, that individually shape community structure, Mantel test to link oomycete patterns with geographical distance and vegetative community structure, DistLM (distance‐based linear models (Legendre & Anderson, 1999)) to test the variation explained by a suite of environmental variables and dbRDA (distance‐based redundancy analysis) to illustrate the linkages between environmental factors identified as strong predictors of community patterns in DistLM using constrained ordination. All analyses were based on Bray–Curtis dissimilarities within the PRIMER software package version 7.0.13 (Primer‐E). PERMANOVA was particularly chosen due to its ability to deal with unbalanced designs applying type III of sums of squares (Anderson et al., 2008). Covariance between normalized continuous variables was assessed using draftsman plots (Clarke & Ainsworth, 1993) applying *ρ* > .8 (Freedman & Zak, 2015). This resulted in removal of C, C/N, and solar radiation from the possible environmental variables. DistLM was initially used to analyze marginal (individual) effects of each variable followed by application of the “best” model building process with adjusted *R* ^2^ selection (Freedman & Zak, 2015) to identify the best set of factors explaining the variation in the community. Site‐specific patterns were illustrated using box and scatter plots prepared in R (R Core Team, 2015).

## 3 RESULTS

### 3.1 Abiotic and biotic environmental conditions

**Figure 3:**
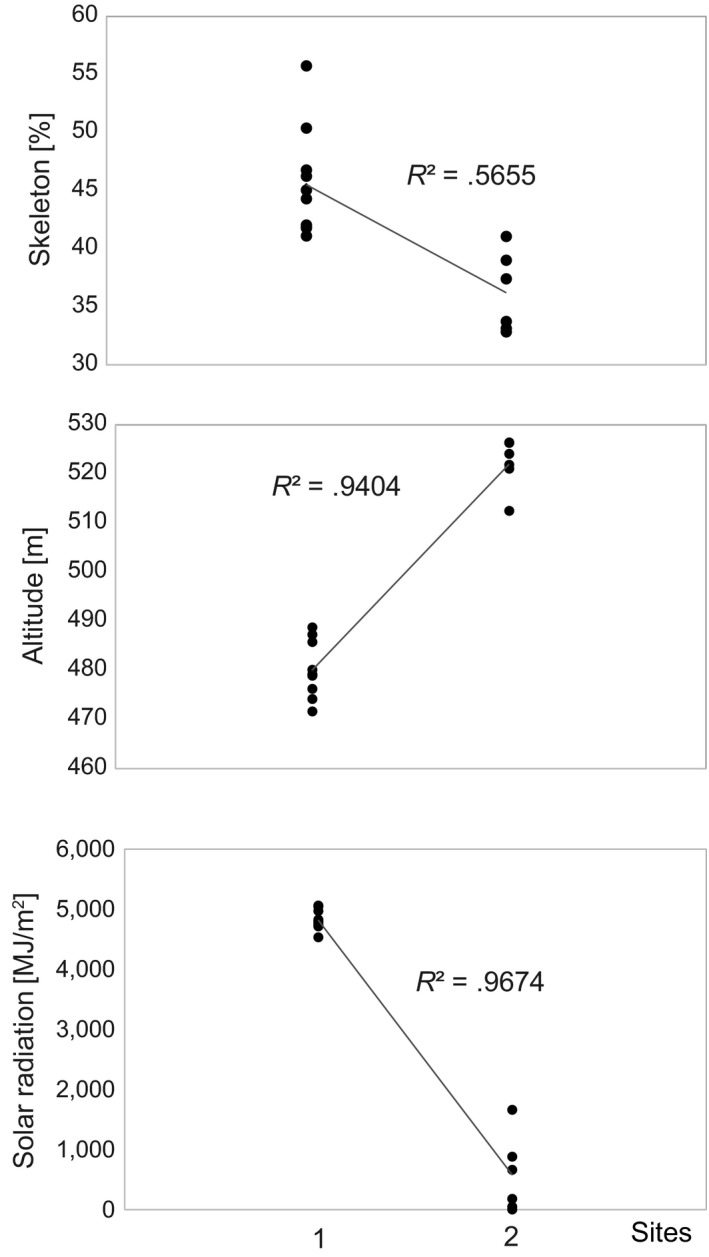
Site‐specific differences for the variables soil skeleton (top), altitude (middle), and solar radiation (bottom) displaying strong linear relationships

Between the two sites sampled, the factors altitude and solar radiation differed significantly. Altitude ranged from 470 to 530 m asl with an average of approximately 480 m asl at site 1 and an average of approximately 520 m asl at site 2. Also, mean annual solar radiation showed distinct differences between the sites varying between 4,820.2 MJ/m^2^ on average in site 1 and 572.5 MJ/m^2^ on average in site 2. Both variables displayed a strong linear relationship (Figure 3) which also applied to soil skeleton albeit to lesser degree. Slope varied from 2° to 22° with an average of approximately 13°, whereas aspect was on average around 166° ranging between 4° and 356°.

The range of soil pH beneath sampled oak trees varied from strong acid (pH 4.0–4.9), slightly acid (pH 5.0–5.9) to weak acid (pH 6.0–6.9). Soil organic C content revealed a large variability (14.8–64.7 g/kg), whereas the ratio of organic C to total N was quite homogeneous and narrow (10–14) indicating a good N supply.

Oak crown foliation varied between 5% and 100%, with an average crown foliation of 62%. The factor tree vigor differed from 6.32 to 76.92, whereas litter cover was on average 11.59%, ranging from 0.5% to 34%. Bare soil ranged from 0.3% to 70% (mean 29.4%). Litter decomposition measurements revealed a mean TBI_k of 0.015 that was accompanied by an average of 0.363 for TBI_S. Vegetation Shannon diversity ranged from 0.73 to 2.41 (Shannon(V)).

### 3.2 Oomycete communities

Taxonomic richness varied between samples, ranging from 1 to 10 OTUs (Table S3). The number of OTUs was highest in samples COIVS9 and COI14 (10 and 9 OTUs each, respectively), whereas Shannon diversity was highest in samples HOII6 and COIVS9 (2.44 and 2.34, respectively). Goods coverage indicated a high coverage of present diversity ranging from 0.995–1.000 (Table S3).

**Figure 4:**
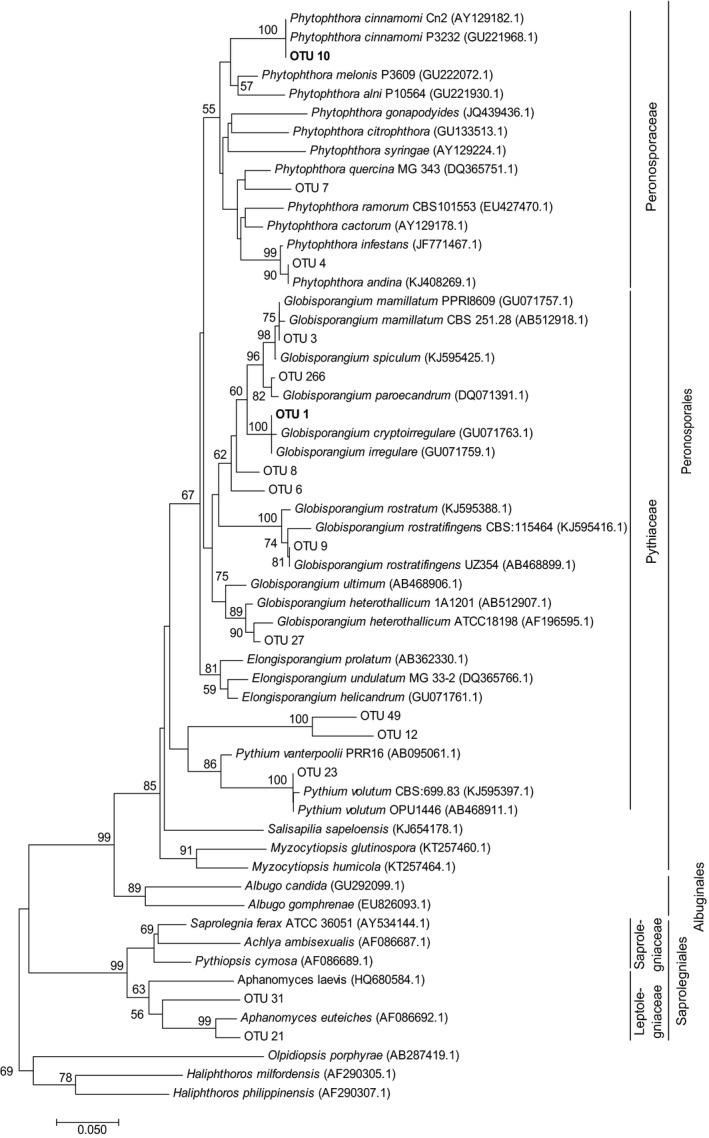
Neighbor‐joining tree of cox2 sequences of 15 OTUs detected in association with oak roots. Reference sequence names are given followed by the GenBank accession number. Bootstrap values >50% are displayed. OTU 1 and OTU 10 showing strong site‐specific occurrences are highlighted in bold

Members of the Peronosporales dominated the oomycete communities (81%), but also members of the Saprolegniales were detected (19%) (Figure 4). Most members of the Peronosporales belonged to the Pythiaceae, specifically to the genera *Globisporangium* (38%) and *Pythium* (23.8%) (Figure 4). Within the Peronosporales, three members of the Peronosporaceae, genus *Phytophthora* were detected. Within the Saprolegniales, members of Leptolegniaceae were found.

The most abundant OTU was OTU 1, related to *G. irregulare* (34.9% of all reads) followed by OTU 10, a relative of *P. cinnamomi* and OTU 4, related to *P. andina* (16.7% and 16.6%, respectively). OTU 1 was widespread, occurring in 14 of 16 samples, followed by OTU 4 detected in 11 of 16 samples and OTU 8, related to *Globisporangium* sp., detected in 9 of 16 samples.

### 3.3 Community patterns and linkage to environmental characteristics

**Figure 5:**
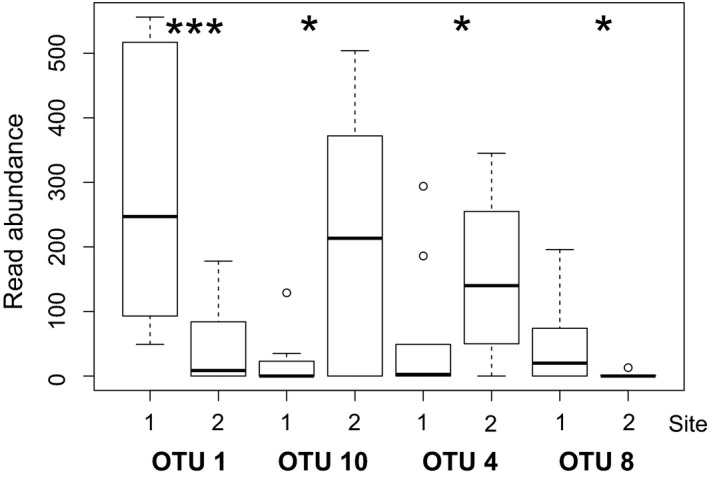
Boxplot showing abundances for OTU 1 and OTU 10 at sites 1 and 2. Significance levels identified by Kruskal–Wallis tests are depicted as *p < .10, **p < .05 and ***p < .01. OTU 1 and OTU 8 have been identified as relatives of Globisporangium irregulare and Globisporangium sp. OTU 10 and OTU 4 were identified as *Phytophthora cinnamomi* and *P. andina*, respectively

Dispersion of oomycete communities appeared similar between the two sites as indicated by PERMDISP (*F* = 0.00036378, *p*: .991 using deviations from centroid). Differences in the microbial communities between the two study sites were detected (ANOSIM Global R: .359, *p* = .006; PERMANOVA + pseudo‐F: 3.7569, *p* = .013, 942 unique permutations; Figure S1). Strong site effects were specifically found for OTUs 1 and 10 (Kruskal–Wallis, *F*: 6.7865, *p* = .009185; *F*: 3.6571, *p* = .05583, respectively) with OTU 1 prevalent at site 1 and OTU 10 predominately at site 2 (Figure 5). A decrease in community similarity over distance was observed based on an increase in Bray–Curtis dissimilarity linked to geographical distance (Figure S2), supported by results of a Mantel test (*ρ*: .226, *p* = .017).

**Figure 6:**
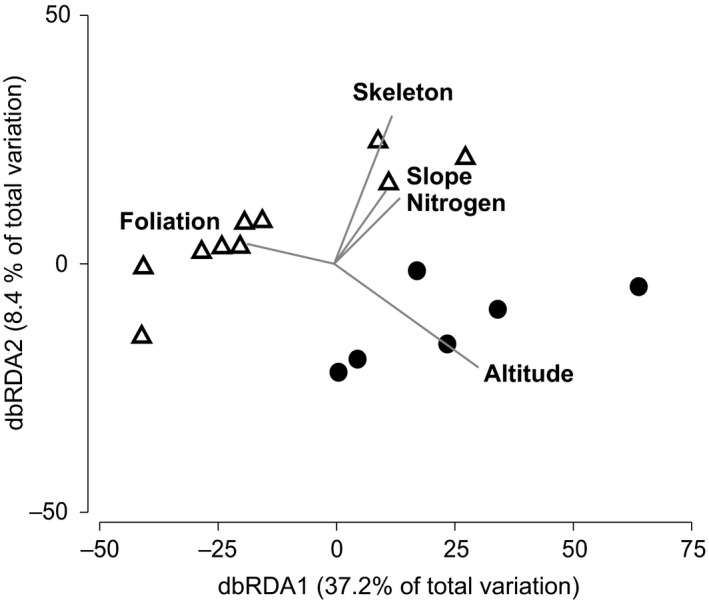
dbRDA biplot based on relative oomycete abundances. Triangles depict samples from site 1, circles display samples from site 2. Displayed are variables with best adjusted *R* ^2^, namely altitude, foliation, nitrogen, skeleton, and slope

Tree species or transparency class (a coarse proxy for symptoms of Iberian oak decline) did not correlate significantly with the oomycete communities tested with ANOSIM and PERMANOVA+. We also did not find a significant correlation between oak (host) species and the distribution of oomycetes. However, strong correlations were observed between oomycete community structure and the variables: altitude, tree vigor, and crown foliation based on marginal tests carried out via DistLM (Table 1). The best combination of variables explaining the observed variation in microbe communities consisted of the variables: altitude, crown foliation, slope, as well as soil skeleton and nitrogen (Table 2). More specifically, a positive correlation was detected between altitude and samples from site 2, whereas the majority of site 1 samples were positively correlated with crown foliation as illustrated in distance‐based redundancy analysis (Figure 6). Three communities of site 1 were positively correlated with the factors soil skeleton and nitrogen as well as slope (Figure 6). Overall, dbRDA axes 1–4 explained 54.15% of variation, whereas axes 1 and 2 explained 45.6% of total variation (Figure 6).

**Table 1:** Correlations between environmental variables and oomycete community patterns displaying marginal DistLM results with environmental variables showing significant correlations at *p* < .10 level in bold

Environmental variables	Marginal tests	
	Pseudo‐F	Proportion of variance explained (%)
**Altitude (m asl)**	**3.28**	**18.96**
**Tree vigor**	**2.08**	**12.95**
**Crown foliation (%)**	**1.90**	**11.94**
Soil N (g/kg)	1.11	7.36
Soil skeleton (%)	0.95	6.37
Bare ground cover (%)	0.72	4.91
Litter stabilization factor	0.71	4.84
Litter cover (%)	0.67	4.56
Aspect (°)	0.52	3.56
Slope (°)	0.51	3.49
Soil pH	0.39	2.71
Litter decomposition rate	0.17	1.21

**Table 2:** Results of the DistLM model using the best model selection procedure and the adjusted *R* ^2^ selection criterion to illustrate correlations between environmental variables and oomycete community patterns

Variables in model	Adjusted *R* ^2^	*R* ^2^
A	.1317	.1896
A, S	.2650	.3630
A, S, N	.2848	.4278
A, S, N, Sl	.3087	.4930
A, S, N, Sl, F	.3144	.5429
A, S, N, Sl, F, V	.3101	.5861

Note

Selected environmental variables included altitude (A), skeleton (S), nitrogen (N), slope (Sl), crown foliation (F) and tree vigor (V). *R* ^2^ shows the proportion of variance explained, whereas adjusted *R* ^2^ provides the *R* ^2^ adjusted for the number of parameters included in the model.

It should be noted that correlations between solar radiation and oomycete community structure were fairly high (best model adjusted *R* ^2^ of .14416 in DistLM), but due to high intercorrelation between solar radiation and the factors altitude and skeleton, this factor was removed from the overall analysis. Although no linkage between oomycete diversity or community structure and Shannon diversity of subcanopy vegetation was detected, vegetative community structure was significantly linked to root oomycete community structure assessed by a Mantel test (*ρ*: .321, *p* = .004).

## 4 DISCUSSION

Based on a newly developed cox2 metabarcoding approach, we were able to detect small scale differences in oomycete communities from the rhizosphere of evergreen oaks in a Spanish oak woodland. To our knowledge, this is the first study to show distance decay in oomycete community structure. Significant associations were observed between oomycetes, host, and habitat characteristics (in particular crown foliation, altitude, slope, soil skeleton, and nitrogen), highlighting the importance of such factors in structuring these oomycete communities. Since members of the Pythiaceae were major components in this habitat, our study complements existing studies that have focussed mainly on the genus *Phytophthora*.

### 4.1 Oomycete community profiling using cox2 Illumina sequencing

A fairly broad taxonomic diversity of oomycetes was detected at two different sites in a Mediterranean oak woodland in Andalusia, Spain. The level of diversity related to the genus *Phytophthora* was comparable to other studies analyzing the rhizosphere of *Q. ilex* or *Q. suber* (Català et al., 2017; Linaldeddu et al., 2014). However, we detected a much higher diversity of Pythiaceae compared to previous research (Lehtijärvi et al., 2017). Furthermore, we detected species belonging to the Leptolegniaceae, not recovered in previous studies.

We found many Peronosporales that are close relatives of known woody host pathogens. Within the Pythiaceae, members of the genus *Globisporangium*, including *G. rostratifingens* originally isolated from Quercus leaf litter (De Cock & Lévesque, 2004) and *Vitis vinifera* (McLeod et al., 2009), were detected. Other taxa within this genus are described to have fairly broad host ranges, such as *G. heterothallicum* found on *Vitis vinifera* (McLeod et al., 2009), *G. irregulare* and *G. paroecandrum* described as pathogens of olive (Sánchez‐Hernández, Ruiz‐Dávila, & Trapero‐Casas, 1997) and apple (Gadgil, Dick, Hood, & Pennycook, 2005; Shivas, 1989) and *G. mamillatum*, a known pathogen of apple (Lévesque, Harlton, & de Cock, 1998). Furthermore, *G. spiculum* has been described to be a root pathogen of *Q. ilex* and *Q. suber* underscoring the importance of the Pythiaceae in the rhizosphere of these hosts (De Vita et al., 2013; Romero et al., 2007). *G. spiculum* is closely related to *G. mamillatum* (Paul et al., 2006), also detected in our study, possibly indicating that *G. mamillatum* occupies a similar niche in the oak rhizosphere. Further studies need to investigate the role of this taxon in oak decline. Pathogenicity of specific Pythiaceae on *Q. suber* and *Q. ilex* was shown to be comparable to *P. cinnamomi* (Belbahri et al., 2005) further underscoring the need to define their link to Iberian oak decline. Furthermore, detected members of the genus *Pythium* are relatives of *P. volutum* described as pathogenic on various herbaceous hosts, mainly grasses (Lévesque et al., 1998). In both genera, *Pythium* and *Globisporangium*, OTUs could be assigned to the genus, but not to known species, which might be attributed to the lack of sufficient sequence data for cox2. Another reason for a lack of a close taxonomic match could be that these species are currently undescribed taxa, also deserving further investigation.

The genus *Phytophthora* was represented by three different species: (a) *P. cinnamomi*, a widespread pathogen with a broad host range including *Q. suber* (Brasier et al., 1993; Scanu et al., 2013) and *Q. ilex* (Corcobado, Solla, Madeira, & Moreno, 2013), (b) a relative of *P. quercina* known as pathogen of various oak species including *Q. ilex* (Pérez‐Sierra et al., 2013) and *Q. robur* (Jung et al., 1999), and (c) *P. andina*, a sister lineage of *P. infestans* known to infect Solanaceae including tree tomato (Forbes, Gamboa, Lindqvist‐Kreuze, Oliva, & Perez, 2016) but also few other non‐Solanaceae hosts (Erwin & Ribeiro, 1996). Plant pathogens belonging to the Saprolegniales included relatives of *Aphanomyces* including the root pathogen *A. euteiches* (Cannesan et al., 2011). Future studies should aim to isolate these oomycetes from the oak rhizosphere and test their impact on these trees.

Our findings show that approaches independent of isolation like metabarcoding or metagenomics can complement classical methods for the detection of key players in oomycete–host interactions. Although the use of mitochondrial genes can be problematic due to uniparental inheritance (Birky, 1995; Martin et al., 2014), the cox2 gene has several advantages for oomycete detection compared to other approaches, given its good taxonomic resolution and suitability as universal barcode for oomycetes (Choi et al., 2015; Martin & Tooley, 2003). To further improve the utility of this barcode, the continual development of corresponding databases is required to overcome the pitfall of many molecular markers: the availability of well‐curated reference sequences (Kang et al., 2010). However, metabarcoding of oomycetes already offers a good approach to study their patterns and distribution of diversity, independent of baiting and isolation procedures.

### 4.2 Linkages with biotic characteristics

No associations were found between tree health classification (on a scale from 1 to 4) and the presence of specific oomycetes, which has also been the case for different *Phytophthora* species in French oak forests (Hansen & Delatour, 1999). However, when the individual degree of crown foliation was used, a stronger association with oomycete community structure was detected. A more quantitative assessment of the host's health was necessary to uncover patterns in the distribution of oomycete taxa. We could also detect an association between subcanopy vegetation and overall oomycete community patterns. A similar association between subcanopy vegetation has been reported in *Quercus* forests for a few soil‐borne oomycete pathogens (Gómez‐Aparicio et al., 2012). The role of subcanopy vegetation as reservoir for oak rhizosphere oomycetes needs to be investigated further.

The collection of more specific data on the root samples collected such as weight, lesion area, and specific sampling depth would allow an even better evaluation of the habitat characteristics favouring specific oomycetes’ distribution.

### 4.3 Linkages with abiotic characteristics

The similarity of oomycete communities at the two sites was affected by geographic distance to a low degree as shown by a Mantel test. This pattern might imply the existence of strong environmental gradients, dispersal limitations, or niche differences between the sites studied, but further sites in the study area should be included to comprehensively investigate whether oomycete communities display distance decay patterns. Our two sites differed in many respects, in particular in altitude, solar radiation, and soil skeleton content. Fungal endophytes also display clear altitudinal patterns for the major species detected (Goldmann et al., 2016; Osono & Hirose, 2009; Siddique & Unterseher, 2016). Similarly, altitude and oak cover were identified as the strongest predictor variables for the distribution of *P. cinnamomi* in Andalusia (Duque‐Lazo, Navarro‐Cerrillo, van Gils, & Groen, 2018) demonstrating the interplay between abiotic and biotic factors for oomycete dispersal.

The link of oomycetes to differences in solar radiation is generally in line with previous findings (Joaquin Duque‐Lazo et al., 2018). However, in our dataset, higher solar radiation was detected in site 1, corresponding with higher levels of *G. irregulare* (OTU 1) but not *P. cinnamomi* (OTU 10). These signatures further support the finding that oomycete community patterns were strongly linked to site‐specific factors including spatial differences in soil properties mainly detected for soil skeleton. Further research needs to confirm these patterns on larger spatial scales and take into account temporal dynamics.

A link between Iberian oak decline and soil factors has been shown previously (Otieno et al., 2006; de Sampaio e Paiva Camilo‐Alves et al., 2013). In their synthesis, oak decline was strongly linked to soil depth and soil compaction, both factors related to water stress (de Sampaio e Paiva Camilo‐Alves et al., 2013). Although these factors were not assessed in our study, we indirectly captured soil depth, as this factor is typically negatively correlated to soil skeleton content and slope (Linstädter & Baumann, 2013). Future studies should investigate the relationship between water stress and oomycete communities in these ecosystems, given the importance of water availability for zoospore movement and infection success (de Sampaio e Paiva Camilo‐Alves et al., 2013; Sena, Crocker, Vincelli, & Barton, 2018). Similarly, the strong link to soil skeleton was not surprising given that higher soil skeleton content is linked to lower soil water holding capacity (directly via a reduced volume of fine material and indirectly via its inverse relationship to soil depth) and that soil moisture is a prerequisite for the spread of oomycetes like *P. cinnamomi* (Hardham & Blackman, 2018). Similarly, the effect of slope is most likely attributable to lower soil moisture (Augenstein, Goeppert, & Goldscheider, 2015; Duque‐Lazo et al., 2018; van Schaik, 2009). Interestingly, we did not find a strong association between pH and oomycete communities as previously observed for different *Phytophthora* species (Jung et al., 2000) and plant‐associated protist diversity in general (Dupont, Griffiths, Bell, & Bass, 2016). Since pH was identified as “master switch” (Glassman et al., 2017) for other kingdoms like fungi, future studies could investigate oomycete communities on larger scales, including a broader range of pH than present at our study sites.

Future studies should also incorporate additional taxonomic groups of microorganisms, since it is known that they may also contribute to plant health and/or microorganism diversity. For example, in *Q. ilex* stands, the presence of *P. cinnamomi* along with the loss of ectomycorrhizal symbiosis for trees was associated with the decline process (Corcobado, Vivas, Moreno, & Solla, 2014). In some cases, fungi have also been associated with oak decline (Linaldeddu et al., 2014), which might explain the unsuccessful oomycete metabarcoding of some roots sampled from trees showing reduced transparency. Microbe–microbe interactions also may influence patterns of oomycete diversity as reported by Sena et al. (2018).

## 5 CONCLUSION

The development of a novel metabarcoding approach based on the cox2 gene enabled the detection of fairly diverse oomycete communities associated with the oak rhizosphere. Both biotic and abiotic factors were associated with rhizosphere oomycete patterns, implying that these micro‐eukaryotes are controlled by similar principles as plant‐associated bacteria (Müller, Vogel, Bai, & Vorholt, 2016) and fungi (Aleklett & Hart, 2013). As for other important plant‐associated kingdoms, future research needs to disentangle ecological mechanisms governing rhizosphere‐associated communities. The identification of such underlying mechanisms of dispersal limitation and environmental filtering would not only help us to better understand rhizosphere oomycete distribution principles, but would also allow us to predict the spread of communities, which in turn would considerably facilitate forest ecosystem management.

## CONFLICT OF INTEREST

None declared.

## AUTHOR CONTRIBUTIONS

GB, AL, LER, MB, MS and TM designed the research. MS conducted the rhizosphere sampling. AL and ALS recorded variables related to the Iberian oak decline (trees’ health class and crown foliation) and other biotic conditions (tree species, age and vigor; litter and bare ground cover; litter decomposition rates; and the floristic composition of the herbaceous layer). TM measured all soil parameters. GW and GB collected and processed geographical data including precise latitude and longitude, altitude, slope and aspect. NT conducted the molecular work and carried out the bioinformatics analyses under supervision of MS. MS and NT carried out the statistical analyses and wrote a first draft of the manuscript, which was further revised by all authors.

## Data Availability

Raw sequence data were deposited in the European Nucleotide Archive under study accession number PRJEB27803 (sample accession numbers ERS2614986–ERS 2615001). The reference sequence database created during this research is available in the Dryad Digital Repository (https://doi.org/10.5061/dryad.56m57q8).
