# Supplementary figures and images for: Site‐specific distribution of oak rhizosphere‐associated oomycetes revealed by cytochrome c oxidase subunit II metabarcoding
Source: Ecol Evol. 2019 Aug 16;9(18):10567–81. doi: 10.1002/ece3.5577 (PMC6787841; doi:10.1002/ece3.5577)

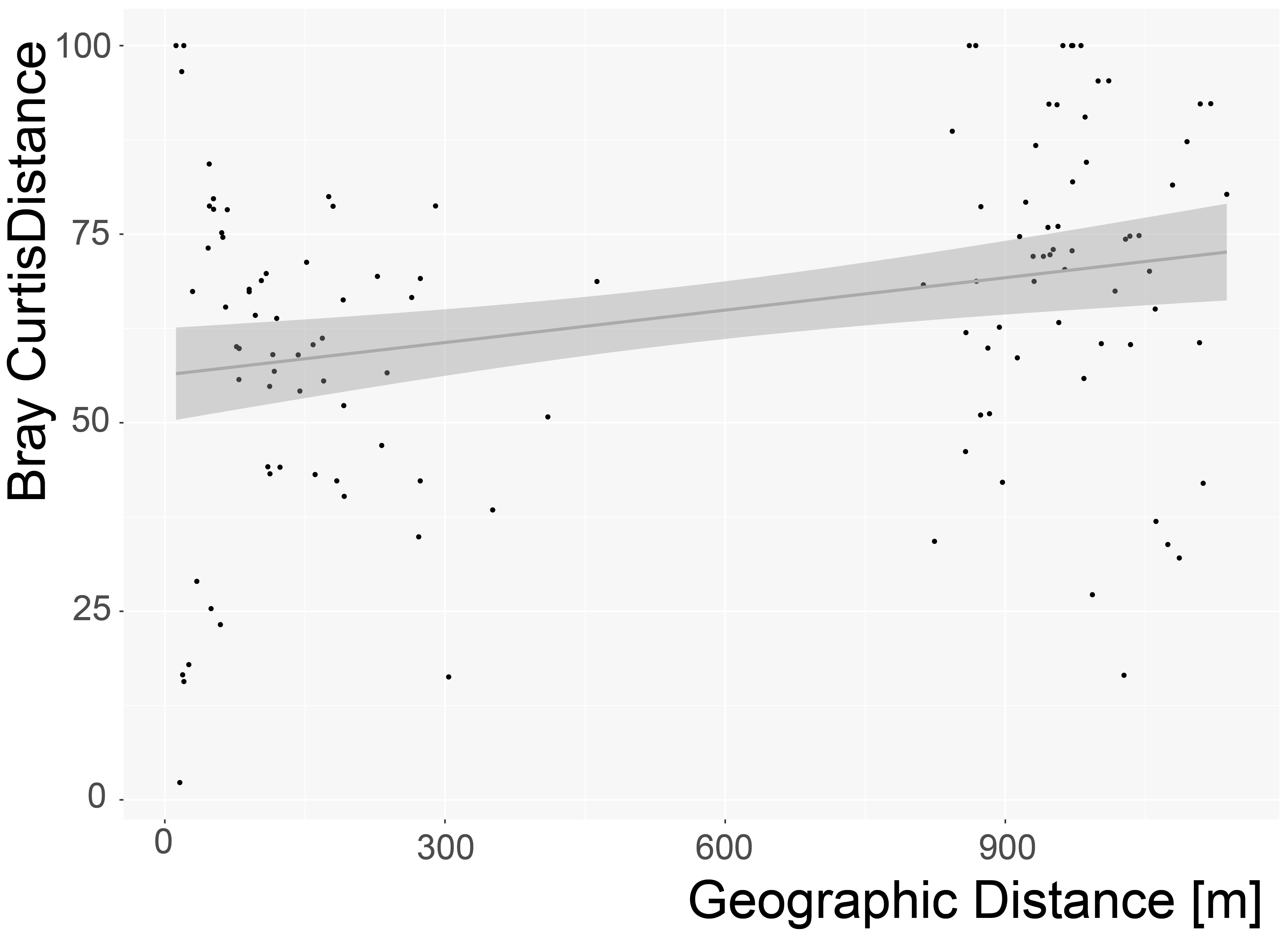

Supplement: Supplementary file 2 [file ECE3-9-10567-s002.jpg]
