# Supplementary material for: Site‐specific distribution of oak rhizosphere‐associated oomycetes revealed by cytochrome c oxidase subunit II metabarcoding
Source: Ecol Evol. 2019 Aug 16;9(18):10567–81. doi: 10.1002/ece3.5577 (PMC6787841; doi:10.1002/ece3.5577)

Table S2. Environmental characteristics of the sites sampled

| ID |  | Longitude | Latitude | Tree Species | Site | Transpa-rency Class | Crown foliation [%] | Tree Vigour | Rwt_HY [MJ/m^2^] | Litter cover [%] | Bare soil [%] | TBI_S | TBI_k | Shannon (V) | Altitude [m] | Slope [degree] | Aspect [degree] | skeleton [%] | pH [-] | N [g/kg] | C [g/kg] | C/N | Soil texture |
| --- | --- | --- | --- | --- | --- | --- | --- | --- | --- | --- | --- | --- | --- | --- | --- | --- | --- | --- | --- | --- | --- | --- | --- |
| COI14 |  | -6.2499 | 37.8732 | Q. suber | 1 | 1 | 100 | 35.98 | 4968.1 | 15 | 65.5 | 0.468 | 0.017 | 1.66 | 488.8 | 17.2 | 234.1 | 45 | 4.45 | 3.4 | 42.3 | 12 | silty sand |
| COII11 |  | -6.2500 | 37.8726 | Q. suber | 1 | 1 | 95 | 37.85 | 4793.3 | 23 | 25.4 | 0.312 | 0.024 | 1.84 | 487.3 | 2.4 | 76.9 | 41 | 4.66 | 4.9 | 64.7 | 13 | silty sand |
| COIII3 |  | -6.2505 | 37.8721 | Q. suber | 1 | 4 | 45 | 22.73 | 5104.7 | 77 | 5.3 | 0.345 | 0.008 | 1.56 | 478.0 | 14.4 | 148.3 | 42 | 4.07 | 3.3 | 47.9 | 14 | silty sand |
| COIV8 |  | -6.2498 | 37.8723 | Q. suber | 1 | 3 | 50 | 11.84 | 4530.4 | 0.5 | 23.0 | 0.314 | 0.015 | 1.08 | 479.1 | 17.1 | 83.2 | 42 | 4.94 | 2.0 | 19.9 | 10 | loamy sand |
| CYII16 |  | -6.2490 | 37.8722 | Q. suber | 1 | 2 | 85 | 61.35 | 5060.2 | 15 | 43.2 | 0.321 | 0.018 | 1.27 | 478.9 | 16.8 | 222.6 | 47 | 4.99 | 1.8 | 18.9 | 11 | ND |
| CYIV25 |  | -6.2522 | 37.8726 | Q. suber | 1 | 4 | 40 | 27.78 | 4712.7 | 5 | 9.3 | 0.397 | 0.012 | 1.64 | 485.8 | 2.6 | 6.4 | 42 | 5.49 | 2.0 | 20.0 | 10 | ND |
| HOI57 |  | -6.2518 | 37.8718 | Q. ilex | 1 | 1 | 100 | 48.41 | 5154.9 | 25 | 27.8 | 0.386 | 0.013 | 1.65 | 464.9 | 16.7 | 204.0 | 55 | 5.47 | 2.7 | 32.4 | 12 | silty sand |
| HOII6 |  | -6.2497 | 37.8719 | Q. ilex | 1 | 2 | 90 | 45.24 | 4754.7 | 12 | 37.2 | 0.333 | 0.011 | 1.99 | 474.2 | 17.3 | 104.8 | 50 | 6.27 | 3.0 | 41.3 | 14 | loamy sand |
| HOIII2 |  | -6.2508 | 37.8720 | Q. ilex | 1 | 3 | 60 | 26.67 | 4824.4 | 2 | 46.0 | 0.344 | 0.012 | 1.93 | 476.2 | 12.3 | 253.5 | 44 | 5.05 | 2.9 | 37.2 | 13 | loamy sand |
| HOIV21 |  | -6.2480 | 37.8722 | Q. ilex | 1 | 4 | 5 | 8.79 | 5044.3 | 1 | 12.3 | 0.433 | 0.018 | 2.31 | 471.6 | 10.5 | 204.8 | 56 | 4.43 | 2.2 | 23.4 | 10 | silty sand |
| HYI49 |  | -6.2485 | 37.8731 | Q. ilex | 1 | 1 | 95 | 76.92 | 4728.9 | 3 | 0.3 | 0.455 | 0.015 | 2.24 | 480.1 | 18.2 | 252.5 | 42 | 5.58 | 1.4 | 14.8 | 10 | ND |
| HYII7 |  | -6.2498 | 37.8722 | Q. ilex | 1 | 2 | 90 | 61.45 | 4785.3 | 15 | 45.6 | 0.352 | 0.012 | 2.07 | 479.2 | 13.0 | 98.6 | 46 | 4.38 | 1.7 | 17.5 | 10 | ND |
| HYIII1 |  | -6.2507 | 37.8720 | Q. ilex | 1 | 3 | 50 | 39.27 | 5106.5 | 3 | 96.1 | 0.468 | 0.012 | 1.30 | 476.2 | 10.3 | 190.9 | 46 | 4.38 | 1.6 | 17.9 | 12 | ND |
| COIIIS2 |  | -6.2549 | 37.8653 | Q. suber | 2 | 3 | 70 | 17.91 | 661.5 | 15 | 20.0 | 0.285 | 0.012 | 1.58 | 524.1 | 9.1 | 3.8 | 34 | 4.31 | 2.7 | 30.7 | 11 | silty sand |
| COIIIS3 |  | -6.2543 | 37.8656 | Q. suber | 2 | 3 | 75 | 23.19 | 2859.5 | 10 | 5.0 | 0.375 | 0.013 | 2.15 | 516.0 | 20.9 | 313.7 | 25 | 6.20 | 4.6 | 60.6 | 13 | loamy sand |
| COIIS1 |  | -6.2549 | 37.8652 | Q. suber | 2 | 2 | 80 | 25.88 | 2.0 | 34 | 20.0 | 0.377 | 0.017 | 1.72 | 526.4 | 8.6 | 26.6 | 39 | 5.79 | 3.5 | 41.4 | 12 | silty sand |
| COIIS4 |  | -6.2548 | 37.8656 | Q. suber | 2 | 2 | 90 | 41.87 | 3.2 | 0 | 10.0 | 0.341 | 0.017 | 0.23 | 515.5 | 32.6 | 40.0 | 39 | 5.92 | 5.0 | 72.9 | 14 | loamy sand |
| COIIIS10 |  | -6.2545 | 37.8656 | Q. suber | 2 | 3 | 55 | 18.88 | 181.1 | 15 | 28.0 | 0.259 | 0.010 | 2.28 | 512.5 | 21.9 | 351.1 | 33 | 5.05 | 3.4 | 42.6 | 13 | loamy sand |
| COIVS6 |  | -6.2547 | 37.8652 | Q. suber | 2 | 4 | 5 | 6.32 | 1661.4 | 10 | 70.0 | 0.464 | 0.017 | 2.30 | 526.3 | 13.7 | 354.0 | 41 | 4.48 | 2.6 | 30.4 | 11 | loamy sand |
| COIVS7 |  | -6.2547 | 37.8654 | Q. suber | 2 | 4 | 10 | 9.36 | 855.7 | 5 | 40.0 | 0.342 | 0.008 | 2.03 | 522.6 | 14.7 | 354.4 | 41 | 5.09 | 2.9 | 35.4 | 12 | loamy sand |
| COIVS8 |  | -6.2548 | 37.8654 | Q. suber | 2 | 4 | 25 | 13.22 | 48.0 | 5 | 5.0 | 0.391 | 0.012 | 2.41 | 521.9 | 13.4 | 29.8 | 37 | 4.51 | 1.9 | 18.7 | 10 | loamy sand |
| COIVS9 |  | -6.2553 | 37.8654 | Q. suber | 2 | 4 | 45 | 20.62 | 881.0 | 15 | 20.0 | 0.307 | 0.009 | 0.73 | 521.1 | 16.2 | 356.2 | 33 | 4.84 | 1.9 | 18.7 | 10 | loamy sand |

ND: not determined

Table S3. Sequences after merging and alpha diversity metrics for sequenced and rarefied samples

| **Sample ID** | **Sequences after merging** | **Index 1 I7** | **Index 2 I5** | **No of OTUs** | **Shannon** | **Goods coverage** |
| --- | --- | --- | --- | --- | --- | --- |
| HOIII2 | 10242 | TAGCGCTC | GAGCCTTA | 5 | 1.583 | 1.000 |
| HOII6 | 187465 | TAGCGCTC | TTATGCGA | 8 | 2.437 | 1.000 |
| HYII7 | 56379 | ACTGAGCG | TCGACTAG | 2 | 0.141 | 1.000 |
| COIV8 | 180628 | ACTGAGCG | TTCTAGCT | 1 | 0.000 | 1.000 |
| COII11 | 65875 | ACTGAGCG | CCTAGAGT | 7 | 1.716 | 0.998 |
| COI14 | 84719 | ACTGAGCG | GCGTAAGA | 9 | 1.456 | 1.000 |
| CYII16 | 53721 | ACTGAGCG | CTATTAAG | 6 | 1.622 | 0.995 |
| HOIV21 | 184662 | ACTGAGCG | AAGGCTAT | 5 | 1.727 | 1.000 |
| CYIV25 | 56895 | ACTGAGCG | GAGCCTTA | 4 | 0.447 | 0.998 |
| HYI49 | 220706 | ACTGAGCG | TTATGCGA | 5 | 1.220 | 0.998 |
| COIIS1 | 1376 | TAGCGCTC | TCGACTAG | 3 | 1.013 | 0.998 |
| COIIIS2 | 97032 | TAGCGCTC | TTCTAGCT | 8 | 2.275 | 1.000 |
| COIVS6 | 6813 | TAGCGCTC | CCTAGAGT | 3 | 0.934 | 0.998 |
| COIVS8 | 16835 | TAGCGCTC | GCGTAAGA | 4 | 0.477 | 0.996 |
| COIVS9 | 4679 | TAGCGCTC | CTATTAAG | 10 | 2.336 | 0.998 |
| COIVS10 | 4952 | TAGCGCTC | AAGGCTAT | 3 | 1.327 | 1.000 |

Figure S1: PCA biplot based on relative oomycete abundances, triangles depict samples from site 1, circles display samples from site 2


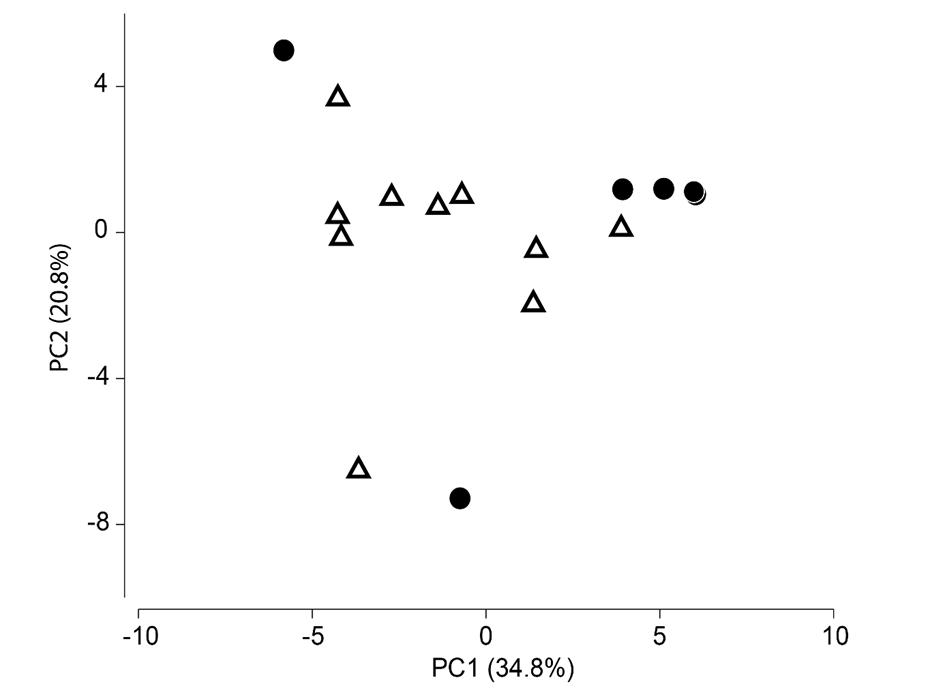


Figure S2: Correlation between geographical distance and Bray Curtis dissimilarity of rhizosphere oomycetes


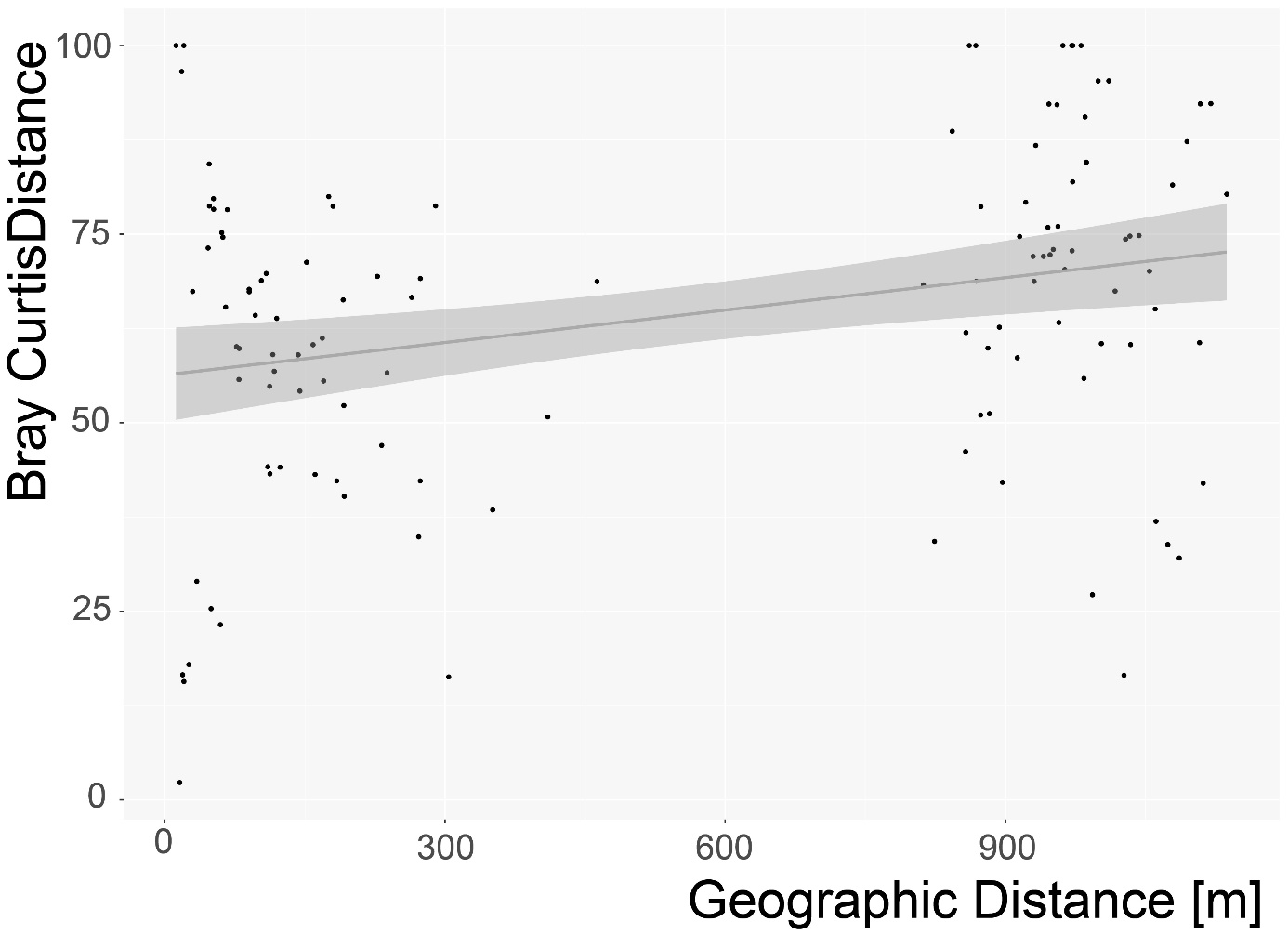

Supplement: Supplementary file 5 [file ECE3-9-10567-s005.docx]
